# Your Boy

**Published:** 1912-12

**Authors:** 


### BUFFALO MEDICAL JOURNAL

**A Monthly Review of Medicine and Surgery**

*EDITOR*

A. L. BENEDICT.

All communications, whether of a literary or business nature, books for review and exchanges, should be addressed to the editor, **228 Summer Street, Cor. Elmwood Avenue, Buffalo, N. Y. Subscribers, advertisers and exchanges please revise address.** Please make personal or telephonic calls before 1 p. m.

The Buffalo Medical Journal will publish regularly. full transactions of any professional organization in western New York at cost. Personal notices, brief reports of society proceedings, and the like, are always welcome and will be published withou charge.

Subscriptions may commence at any time. $2.00 per year in advance.

Vol. LXVIII. DECEMBER, 1912 No. 5

*One excuse is more convincing than several, however good each may be. And an absolutely valid excuse is not so good as getting a thing done in spite of obstacles.*

### Your Boy

Doctor, you are the hygienic guardian of the community, trained to prevent as well as to treat disease. More than other men, you realize the immense economy and efficiency of prophylaxis as compared with therapeutics.

Does your baby need to be circumcised?

Has your boy been vaccinated—and revaccinated after five years?

Can he breathe through his nose?

If he developed diphtheria, could you inspect his throat without making him gag and causing a fit of rage and terror that might result in fatal syncope?

Does he know poison ivy, deadly night-shade, datura, mushrooms, etc.?

Has he enough knowledge of means of infection—or aesthetics—not to use another boy's tooth brush, handkerchief, towel, etc., not to put coins in his mouth, not to drink ditch water, etc.?

Are there cavities in his teeth?

How much and what does he eat? Does he eat all sorts of trash between meals?

Can he swim?

Does he play in the street? Does he realize that he must stop, look and listen, before crossing a railroad track or passing a curb stone?

Is he so sympathetic that he will pick up a sick cat or visit a play-mate confined to the house with scarlet fever?

Do his bowels move daily and, if not, will he tell you about their condition?

If some big boy tries to teach him to masturbate or if he encounters a pervert, does he know his danger?

There are many more questions—ask them yourself.

